# 

**Published:** 1919-01

**Authors:** 


					﻿VOL. XXXIV.
JANUARY, 1919.
No. 7.
Texas
Medical
Journal
1	X	t 1	X
^Ente^d at thelpostofflce at	mgifl matter
				

